# Drunken Women

**Published:** 1868-09

**Authors:** 


### DRUNKEN WOMEN.

"Doing evil that good may come," is a creed in a certain class of minds, as if the Almighty required Satanic aid to carry on his purposes among men, and that temperance lecturer at Springfield, Ohio, who made the specific statement that "thirteen hundred rich men's daughters" had been sent to the Inebriates' Asylum at Binghampton, New York, to be treated for drunkenness," and ever so many doctors and lawyers and ministers, merits the contempt of the civilized world as does every man who utters a malign sentiment against woman, as a class; every paragraph carries the falsehood of a low nature on its very face; why should there be even thirteen hundred rich men's daughters; why not make it 1250 or 1500, and instead of even 600 lawyers, why not make it 692, or forty-one million eight hundred and two thousand nine hundred and seven. How comes it too that no mention is made of poor men's daughters? how does it happen there are no mechanics' daughters there, no politicians', no day laborers'? We really do not think a meaner untruth was ever put in print. The actual fact of the case, as far as women are concerned, is simply this, "a female patient has never been admitted into the asylum named—and never will be; it is for drunken men alone."

